# Periosteal progenitors contribute to load-induced bone formation in adult mice and require primary cilia to sense mechanical stimulation

**DOI:** 10.1186/s13287-018-0930-1

**Published:** 2018-07-11

**Authors:** Emily R. Moore, Ya Xing Zhu, Han Seul Ryu, Christopher R. Jacobs

**Affiliations:** Columbia University Department of Biomedical Engineering, 500 W 120th St, New York, NY 10027 USA

**Keywords:** Primary cilium, Periosteal progenitors, Osteoblasts, Osteochondroprogenitors, Mechanotransduction, Bone regeneration, Prx1

## Abstract

**Background:**

The fully developed adult skeleton adapts to mechanical forces by generating more bone, usually at the periosteal surface. Progenitor cells in the periosteum are believed to differentiate into bone-forming osteoblasts that contribute to load-induced adult bone formation, but in vivo evidence does not yet exist. Furthermore, the mechanism by which periosteal progenitors might sense physical loading and trigger differentiation is unknown. We propose that periosteal osteochondroprogenitors (OCPs) directly sense mechanical load and differentiate into bone-forming osteoblasts via their primary cilia, mechanosensory organelles known to be involved in osteogenic differentiation.

**Methods:**

We generated a diphtheria toxin ablation mouse model and performed ulnar loading and dynamic histomorphometry to quantify the contribution of periosteal OCPs in adult bone formation in vivo. We also generated a primary cilium knockout model and isolated periosteal cells to study the role of the cilium in periosteal OCP mechanosensing in vitro. Experimental groups were compared using one-way analysis of variance or student’s *t* test, and sample size was determined to achieve a minimum power of 80%.

**Results:**

Mice without periosteal OCPs had severely attenuated mechanically induced bone formation and lacked the mineralization necessary for daily skeletal maintenance. Our in vitro results demonstrate that OCPs in the periosteum uniquely sense fluid shear and exhibit changes in osteogenic markers consistent with osteoblast differentiation; however, this response is essentially lost when the primary cilium is absent.

**Conclusions:**

Combined, our data show that periosteal progenitors are a mechanosensitive cell source that significantly contribute to adult skeletal maintenance. More importantly, an OCP population persists in the adult skeleton and these cells, as well as their cilia, are promising targets for bone regeneration strategies.

**Electronic supplementary material:**

The online version of this article (10.1186/s13287-018-0930-1) contains supplementary material, which is available to authorized users.

## Background

Bone is a dynamic, self-repairing tissue, and its adaptation is of interest for developing regenerative therapeutics. One commonly studied natural phenomenon is adult bone formation, whereby the mature skeleton generates more bone in response to heightened physical forces. The leading paradigm is that mechanosensitive osteocytes sense mechanical loading and secrete paracrine factors to recruit osteogenic precursors for new bone formation [1]. Conversely, a lack of physical loading causes osteocytes to trigger osteoclastic bone resorption, adapting the skeleton to reduce weight and metabolic demands while maintaining sufficient strength to withstand the reduced loads. Treatments that prevent resorption to combat bone loss have been associated with atypical fracture [2, 3]. Thus, the focus has shifted towards strategies favoring bone anabolism. Investigating adaptation to increased loading provides insights into the recruitment of osteogenic precursors, as well as activation of osteoblasts to deposit new bone matrix. Ideal regenerative techniques to generate bone where it is needed would combine an effective osteoprogenitor cell source with agents to enhance cell-mediated mineral apposition. As a result, determining the origin of osteogenic precursors and elucidating the mechanisms that trigger osteoblast activity will significantly benefit emerging therapeutics.

The periosteum, a thin coating surrounding the bone, has recently re-emerged as an attractive source of osteogenic progenitor cells. This tissue can be isolated from several locations in the body, such as the anterior tibia and the spinous process. Periosteum is easier to extract than bone marrow mesenchymal stem cells (MSCs), and its inner cambium layer is rich in progenitors believed to differentiate into bone-forming osteoblasts [4–6]. Furthermore, load-induced bone formation is favored over resorption at the periosteal surface, suggesting periosteal progenitors are biased towards osteogenesis [7]. In general, mechanical stimulation is thought to elicit an osteogenic response from progenitor cells [8, 9] and, indeed, osteogenic markers are upregulated in mechanically strained periosteal cells [10]. Despite the mounting evidence suggesting osteoblasts arise from the periosteum during bone formation, the exact mechanism for how periosteal cells respond to heightened loads remains unknown.

A subset of cells in the postnatal periosteum express Paired related homeobox 1 (Prx1) and preferentially differentiate towards an osteogenic or chondrogenic lineage in vitro [11]. These osteochondroprogenitor cells (OCPs) profoundly contribute to adult fracture repair [11, 12], but their role in daily skeletal maintenance and adult bone formation remains unexplored. Prx1 is expressed throughout the mesenchymal limb bud, and the Prx1Cre transgene is believed to label multipotent mesenchymal progenitors during skeletal development [13]. Studies using constitutive Prx1-driven Cre expression identified recombined osteoblasts, osteocytes, adipocytes, perivascular stromal cells, and progenitor cells in many tissues, including the periosteum and bone marrow [11, 14–16]. However, the creators of an inducible Prx1CreER model suggest Prx1 expression is perhaps more restricted than previously reported and may be useful for studying periosteal OCPs [11]. Our previous work with this inducible model indicates the Prx1Cre transgene is present in the periosteum of skeletally mature mice, but Prx1 expression patterns in the adult remain largely uncharacterized.

One possible mechanism for how periosteal OCPs sense mechanical loading is that they directly sense physical stimulation through their primary cilia, sensory organelles known to transduce external stimuli into intracellular signaling cascades. The primary cilium is present on all bone cells and is important for cell differentiation. Specifically, previous in vitro studies in our laboratory suggest cilia are necessary for osteogenic differentiation of periosteal progenitors [4, 9] and human MSCs [8, 17]. Eliminating key ciliary proteins results in attenuated bone formation in vivo [18–20]. Furthermore, we recently determined that mechanical loading activates progenitors to form bone in adults, but this response is attenuated in mice containing a Prx1-driven cilium knockout (Chen et al., manuscript submitted). Despite the clear implications, the mechanism that allows periosteal OCP primary cilia to mediate the response to mechanical stimulation has yet to be investigated.

Periosteal OCPs may also differentiate in response to paracrine signals from mechanically stimulated osteocytes. In response to loading, osteocytes are thought to signal to osteoblasts and osteogenic precursors, triggering progenitor differentiation, osteoblast proliferation, and, ultimately, matrix deposition. Our laboratory and others have demonstrated that conditioned media from osteocytes exposed to fluid shear triggers osteoblast activity [21] and induces osteogenic differentiation of MSCs [8]. In fact, conditioned media from osteocytes in a static environment alone has been shown to encourage osteogenic differentiation of bone marrow stromal cells [22]. Osteoblasts respond directly to mechanical stimulation [23], as well as paracrine signaling from osteocytes [1, 21], but it is unknown whether periosteal OCPs behave similarly. Furthermore, our conditioned media study indicates that the primary cilium is necessary for paracrine signaling between osteocytes and MSCs [8]. If periosteal OCPs do respond to osteocyte signaling, it will be important to know whether this process is mediated by the primary cilium.

The objectives of this study are four-fold. First, to determine Prx1 expression in the skeletally mature adult mouse ulna using a fluorescent reporter model. Spatially mapping the presence of the Prx1Cre-GFP transgene is critical for characterizing the affected cell population in our model. Second, to quantify the contribution of Prx1-expressing cells in load-induced bone formation using a conditional diphtheria toxin A (DTA) cell ablation model. Third, to identify whether periosteal cells respond to mechanical stimulation and/or paracrine signaling from osteocytes in vitro. Finally, we seek to determine the mechanism by which primary cilia potentially mediate the periosteal OCP response to mechanical loading through in vitro fluid-shear studies.

## Methods

### Animal models

All mouse models are based on a C57BL6 background. Prx1CreER-GFP males were bred with Rosa26^tdTomato^ females acquired from Jackson Laboratories (Bar Harbor, ME) to generate a Prx1CreER-GFP;Rosa26^tdTomato(−/+)^ fluorescent reporter model. Prx1CreER-GFP females were bred with Rosa26^DTA^ males acquired from Jackson Laboratories to generate Prx1CreER-GFP;Rosa26^DTA(−/+)^ experimental and Rosa26^DTA(−/+)^ control offspring. Animals used for the ulnar loading experiments did not participate in breeding. Prx1CreER-GFP and Prx1CreER-GFP;Ift88^fl/+^ males were bred with Ift88^fl/fl^ females to generate Prx1CreER-GFP;Ift88^fl/fl^ offspring for primary periosteal cell isolations. Genotype was determined using polymerase chain reaction (PCR) and agarose gel electrophoresis. Primer sequences are available upon request. Animals were housed, maintained, and evaluated for health complications in accordance with IACUC standards. All experiments were approved by the Institute of Comparative Medicine at Columbia University.

### Tamoxifen injections

Tamoxifen (Sigma-Aldrich, St. Louis, MO) was dissolved in corn oil (Sigma-Aldrich) in a shaking incubator at 37 °C to create a 25 mg/mL stock solution stored at 4 °C and protected from light. Fresh tamoxifen solution containing 10% ethanol was prepared daily and delivered via injection to ensure consistent exposure. Skeletally mature 16-week-old adult Prx1CreER-GFP;Rosa26^tdTomato^ mice received a single intraperitoneal injection of 100 mg/kg body weight tamoxifen. Adult Prx1CreER-GFP;Rosa26^DTA^ experimental and Rosa26^DTA^ controls received daily intraperitoneal injections of 75 mg/kg tamoxifen solution for 5 days prior to loading, concurrent with the 3 days of loading, and concurrent with fluorochrome label injections after loading. Injections were performed at the same time each morning in a clean cage placed in a laminar flow hood.

### In vivo ulnar loading

Skeletally mature 16-week-old mice were placed under isofluorane anesthesia and the right forelimbs were anchored between two plates attached to an electromagnetic Enduratec ELF 3220 loading system with feedback control (Bose, Framingham, MA). Following an initial 0.1 N load, a peak compressive axial load of 3 N was applied with a 2 Hz sine wave for 120 cycles/day for 3 consecutive days. The nonloaded left forelimbs served as internal controls. Body weight was measured each time an injection or ulnar loading was administered, and cage activity and anesthesia recovery time were observed to monitor animal health. Mice received subcutaneous injections of 10 mg/kg calcein (Sigma-Aldrich) and 70 mg/kg alizarin red (Sigma-Aldrich) 5 and 9 days following initiation of loading, respectively. All animals were euthanized 15 days after the initiation of loading and prepared for analysis.

### Microcomputed tomography (microCT) and dynamic histomorphometric analysis

Upon sacrifice, loaded and nonloaded ulnae were dissected and stored in 70% ethanol for up to a week. The nonloaded limb of each specimen was imaged by microCT (Scanco vivaCT 80, Scanco Medical AG, Brüttisellen, Switzerland) at 10.4 μm isotropic resolution using scan settings of 55 kV, 145 μA, and 300 ms integration time. Bone lengths were determined from the scout view. Cortical bone analyses were performed at the mid-diaphysis of each ulna and Scanco analysis software was used to determine total bone area, the ratio of bone volume to total volume (BV/TV), bone mineral density (BMD), cortical thickness, the polar moment of inertia (*J*), and the minimum and maximum second moments of inertia (I_min_ and I_max_).

Following the microCT scan, ulnae were gradually dehydrated in an ASP300S tissue processor (Leica, Wetzlar, Germany), infiltrated with methyl methacrylate, and embedded in methyl methacrylate and benzoyl peroxide (Sigma-Aldrich), as described previously [18]. Embedded specimens were sectioned at the ulnar midshaft using a diamond-tip blade and saw (Isomet, North Springfield, VA) and transverse sections were imaged on a Fluoview confocal microscope (Olympus, Shinjuku, Tokyo, Japan). The bone surface, single label, and double label perimeters and double label area were quantified in ImageJ (Broken Symmetry Software) to calculate mineralizing surface/bone surface (MS/BS), mineral apposition rate (MAR), and bone formation rate/bone surface (BFR/BS) [18, 24]. All measurements were taken from the periosteal surface. Nonloaded ulnae values were subtracted from loaded values to determine relative measurements of rMS/BS, rMAR, and rBFR/BS, which represent changes specifically due to mechanical loading.

### Histology and immunocytochemistry

Upon sacrifice, ulnae were dissected and fixed overnight at 4 °C. For histological analysis, specimens were fixed in 10% formalin (Sigma-Aldrich), decalcified, embedded in paraffin, sectioned longitudinally in 5μm increments, and stained with hematoxylin and eosin (H&E) (Sigma-Aldrich) for 10 min and 30 s, respectively. Micrographs were collected with a CKX41 inverted microscope (Olympus) at 20× magnification. To determine whether our ablation model was successful in inducing cell death, ulnae were fixed in 4% paraformaldehyde (Sigma-Aldrich), decalcified, and cryosectioned longitudinally in 5μm increments. GFP was visualized and micrographs were collected with a confocal microscope (Olympus) at 100× magnification.

To detect primary cilia in vitro, isolated and sorted primary periosteal OCPs were seeded on glass bottom dishes (MatTek, Ashland, MA) at 2.5 k per dish 24 h prior to experimentation. Following treatment, cells were fixed in 10% formalin, blocked with 15% goat serum, and incubated in a primary antibody for acetylated α-tubulin acquired from a C3B9 hybridoma line (Sigma-Aldrich), followed by a fluorescent secondary (1:500, Alexa-Fluor 488, Life Technologies). All incubations were conducted at room temperature for 1 h. Micrographs were collected with a confocal microscope (Olympus) at 100X magnification.

### Primary cell isolation

Prx1CreER-GFP and Prx1CreER-GFP;Ift88^fl/fl^ juveniles were sacrificed between 3 and 4 weeks of age and their fore- and hindlimbs were dissected. The skin, fascia, connective tissue, and majority of the muscle surrounding the periosteum was removed using a scalpel and the specimens were placed in sterile phosphate-buffered saline (PBS; Life Technologies, Carlsbad, CA) on ice. The epiphyses and remaining muscle surrounding the periosteum were removed, and the specimens were transferred to fresh cold PBS. In a sterile culture hood, the periosteum was scored with a scalpel, peeled off the bone, cut into 1mm^2^ sections, and placed into fibronectin-coated (Sigma-Aldrich) tissue culture dishes containing minimum essential medium (MEM)α (Life Technologies) supplemented with 10% fetal bovine serum (FBS) and 1% penicillin/streptomycin (P/S; Life Technologies). Tissue sections were incubated at 37 °C for 7–10 days and the resulting primary periosteal cells were passaged onto fresh fibronectin-coated tissue culture dishes. Cells were then passaged or sorted upon reaching 80% confluence.

### Cell culture and sorting

Primary periosteal cells were cultured on fibronectin-coated dishes in MEMα supplemented with 10% FBS and 1% P/S at 37 °C. An Influx cell sorter (BD Biosciences, San Jose, CA) was used to separate osteochondroprogenitors (GFP^+^) from other cells of the periosteum (GFP^–^). Sorted cells were cultured for 1–2 weeks until a sufficient number were available for flow studies. Passages P2 to P5 were used for all in-vitro experiments. MLO-Y4 osteocytes were cultured on collagen type I-coated tissue culture dishes (Corning Inc., Corning, NY) in MEMα supplemented with 5% FBS, 5% calf serum (CS), and 1% P/S at 37 °C. P40 to P45 were used for conditioned media studies.

### In-vitro fluid-flow studies

To test the effects of direct mechanical stimulation on periosteal cells, osteochondroprogenitors (GFP^+^) and other cells of the periosteum (GFP^–^) were seeded on fibronectin-coated glass slides (75 × 38 × 1 mm; Fisher Scientific, Hampton, NH) and cultured in reduced serum media (MEMα supplemented with 2.5% FBS, 2.5% CS, and 0.5% P/S) 48 h prior to application of flow. To disrupt primary cilia, cells were treated with the active metabolite of tamoxifen, (Z)-4-hydroxytamoxifen (Sigma-Aldrich), diluted to 5 μg/mL in 95% ethanol or vehicle control 24 h prior to flow. Upon reaching 80% confluence, primary cells were placed in parallel-plate flow chambers (56 × 24 × 0.28 mm), incubated at 37 °C for 30 min at rest, and exposed to 60 min of oscillatory fluid flow (OFF) at 1 Hz with a peak shear stress of 10 dyn/cm^2^. Slides were removed from the chambers and lysed immediately with TriReagent (Sigma-Aldrich) to isolate RNA. Real-time quantitative PCR (RT-qPCR) was performed to quantify flow-induced changes in Cyclooxygenase-2 (COX-2), Osteopontin (OPN), Runt-related transcription factor 2 (RUNX2), Bone gamma-carboxyglutamic acid-containing protein (BGLAP), and Glyceraldehyde 3-phosphate dehydrogenase (GAPDH) using fluorescent primers (Life Technologies) and an ABI PRISM 7900 (Applied Biosystems, Foster City, CA). Samples were performed in triplicate and normalized to the expression of GAPDH, a housekeeping gene. OFF samples were normalized to static controls.

For conditioned media studies, MLO-Y4 osteocytes were seeded on four-well plates (Fisher Scientific, 127.8 × 85.5 mm) at 75k cells per well and cultured for approximately 2 days until cells were 80% confluent. Isolated primary Prx1CreER-GFP;Ift88^fl/fl^ periosteal OCPs were seeded on four-well dishes at 100k per well and treated with (Z)-4-hydroxytamoxifen or vehicle control for 24 h prior to receiving conditioned media. Fresh media (3.5 mL) was placed on MLO-Y4s immediately before being exposed to OFF (0.78 dyn/cm^2^, 0.33 Hz) on a platform rocker [8] or static conditions at 37 °C for 12 h. At the conclusion of flow, media were aspirated from MLO-Y4s and centrifuged at 2 rpm for 10 min to prevent contamination by detached MLO-Y4s; 2 mL of centrifuged media was transferred to each well of the primary periosteal cells, which were cultured for 48 h and lysed for RT-qPCR as described previously.

### Statistics

Animals were randomly assigned to groups depending on genotype, and researchers were blinded to all data analysis. No sex-dependent differences were identified according to a two-way analysis of variance (ANOVA), and so males and females were grouped together for dynamic histomorphometry and microCT analysis. We could not assume normality for our in vivo data and so comparisons were determined with a one-way ANOVA and Bonferroni post-hoc correction. Our in vitro data satisfied conditions of normality and so we analyzed comparisons using a two-tailed student’s *t* test. Values are reported as mean ± SEM, with *p* < 0.05 considered statistically significant. The sample size was selected to achieve a power of at least 80% for all tests.

## Results

### Prx1 expression is restricted to the periosteum in ulnae of skeletally mature adults

**Fig. 1:**
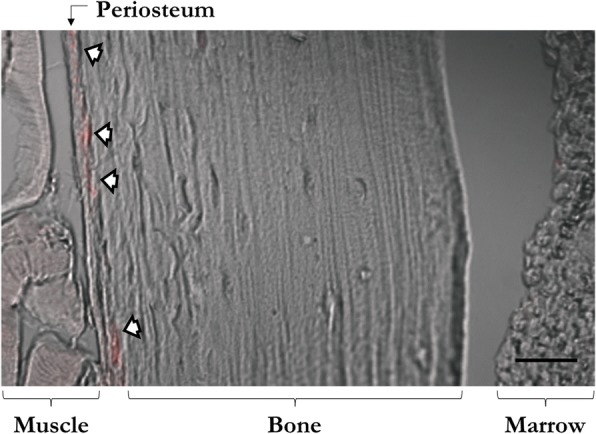
Prx1 expression is confined to the adult periosteum in the ulnar midshaft. Skeletally mature 16-week-old Prx1CreER-GFP;Rosa26^tdTomato^ mice received a single dose of tamoxifen to induce tdTomato production and were sacrificed a week later. Recombined cells (red) were found in the periosteum (white arrows) of the ulna, but were absent from cortical bone, trabecular bone, muscle, and bone marrow. Micrographs were captured with a confocal microscope at 20X. Scale bar = 50 μm

Skeletally mature 16-week-old fluorescent reporter mice (Prx1CreER-GFP;Rosa26^tdTomato^) were injected with a single dose of tamoxifen to determine the location of Prx1-expressing cells and confirm Cre activity. Red fluorescent cells were indeed present, indicating successful Cre recombination. We found recombined cells in the ulnar periosteum (Fig. 1) and perichondrium (not shown), but these cells were absent from cortical bone, trabecular bone, muscle, and marrow. Therefore, it can be concluded that the Prx1CreER-GFP model is an appropriate tool for determining the effects of periosteal OCP ablation on load-induced adult bone formation.

### Tamoxifen treatment successfully ablates periosteal progenitors in vivo and primary cilia in vitro

**Fig. 2:**
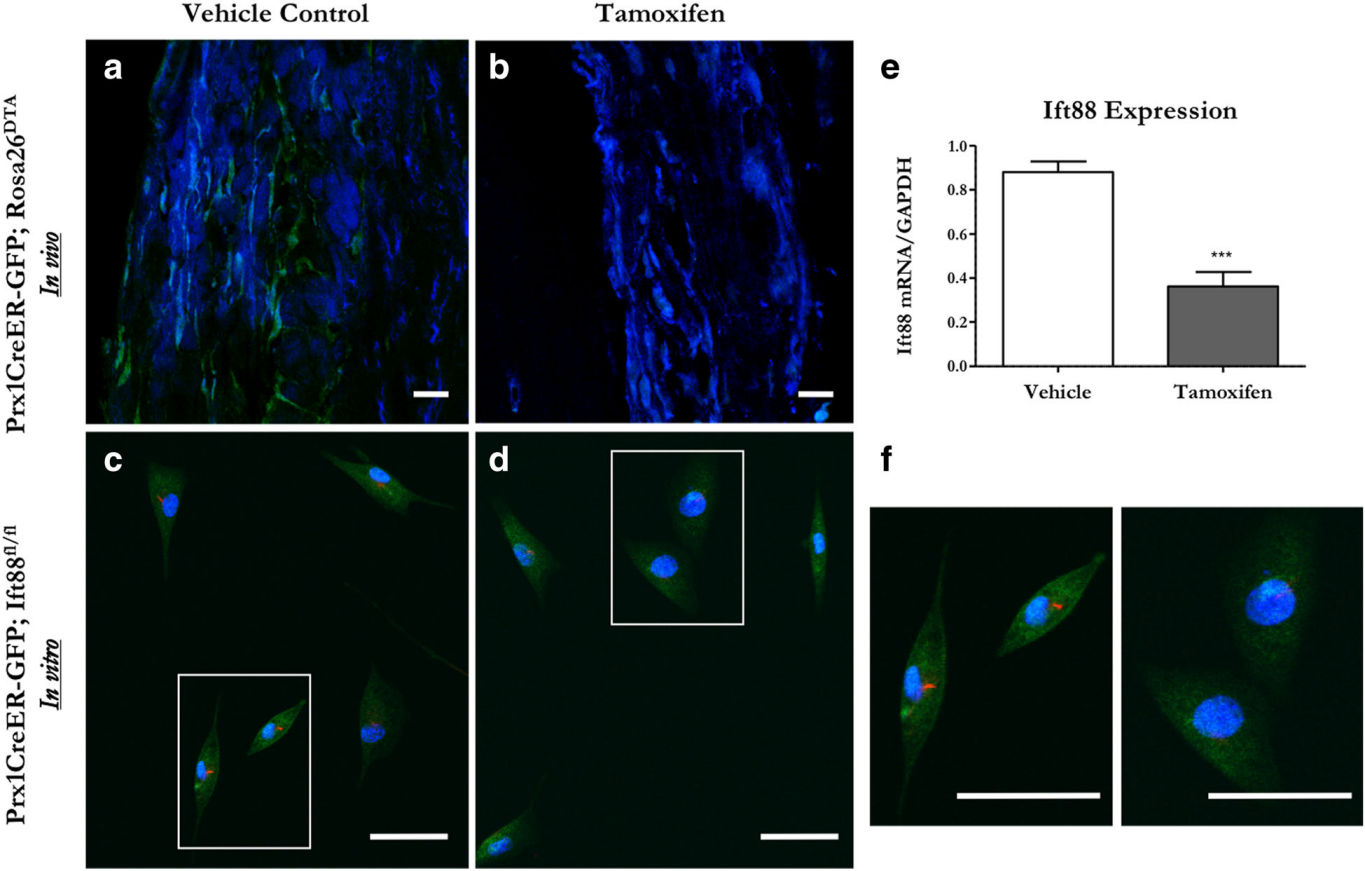
Tamoxifen treatment successfully ablates periosteal progenitors and their cilia in our transgenic animal models. Skeletally mature 16-week-old Prx1CreER-GFP;Rosa26^DTA^ ablation mice and Prx1CreER-GFP controls received tamoxifen injections and were sacrificed a week later. Prx1-expressing cells (green) were observed in the periosteum of Prx1CreER-GFP control ulnae (**a**) but were absent in Prx1CreER-GFP;Rosa26^DTA^ animals (**b**). Primary OCPs obtained from 3-week-old Prx1CreER-GFP;Ift88^fl/fl^ animals (**c**) normally contain primary cilia (red); however, the presence and length of these cilia was diminished after exposure to 5 μM 4-hydroxytamoxifen (**d**). Indeed, cells treated with tamoxifen have decreased Ift88 mRNA expression (**e**). A magnified view of box region in panels c and d (**f**). Micrographs of the periosteum and isolated cells were collected at 20X and 40X, respectively. Nuclei are displayed in blue. Scale bars = 50 μm. Data are reported as mean and standard error. *n* = 4 for each group, ***p < 0.0001

We generated inducible ablation and primary cilium knockout (KO) models to evaluate the effects of eliminating OCPs and their primary cilia. Animals did not exhibit any noticeable adverse effects from tamoxifen injection or OCP ablation. We visualized GFP expression to determine whether Prx1-expressing OCPs were present in the periosteal tissue of Prx1CreER-GFP;Rosa26^DTA^ mutant and Prx1CreER-GFP control adult mice injected with tamoxifen. Control animal periosteum contained green fluorescent cells but these were absent from mutant periosteum (Fig. 2), indicating successful ablation of periosteal OCPs in vivo. We then determined whether tamoxifen treatment could be used to disrupt periosteal OCP cilia in vitro. Primary OCPs isolated from the periosteum of Prx1CreER-GFP;Ift88^fl/fl^ juveniles were sorted via GFP and treated with 4-hydroxytamoxifen, the active metabolite of tamoxifen, to induce an Ift88-mediated primary cilium knockout. Indeed, cells treated with 4-hydroxytamoxifen had nearly half the Ift88 mRNA expression observed in vehicle controls (Fig. 2). Furthermore, these cells demonstrated shorter and fewer cilia compared with vehicle controls (Fig. 2), confirming that this treatment successfully disrupted primary cilia in vitro.

### Bone formation is severely attenuated in mice lacking periosteal progenitors

Genetic modifications may alter bone structure in transgenic animals, resulting in changes that influence the skeleton’s response to load. To ensure that our ablation model did not alter bone properties and introduce a confounding variable in our loading studies, we first assessed bone microstructure in our Prx1CreER-GFP;Rosa26^DTA^ and Rosa26^DTA^ animals using microCT. We did not identify any differences in bone microstructure (Table 1); therefore, our dynamic histomorphometry results depict changes in bone formation specifically due to periosteal OCP ablation prior to loading.

**Table 1:** Ulna cortical bone microarchitecture

Parameter	Female		Male	
	Control	Ablation	Control	Ablation
*n*	5	5	8	5
Bone area (mm^2^)	0.281 ± 0.012	0.263 ± 0.018	0.294 ± 0.005	0.295 ± 0.006
Cortical thickness (mm)	0.172 ± 0.001	0.169 ± 0.004	0.169 ± 0.004	0.175 ± 0.002
BV/TV (%)	0.867 ± 0.002	0.864 ± 0.003	0.863 ± 0.002	0.863 ± 0.001
BMD (mg/cm^3^ of HA)	1293 ± 5.3	1289 ± 5.2	1282 ± 3.3	1288 ± 7.0
J (mm^4^)	0.024 ± 0.003	0.020 ± 0.003	0.027 ± 0.001	0.027 ± 0.001
I_max_ (mm^4^)	0.019 ± 0.003	0.016 ± 0.003	0.022 ± 0.001	0.023 ± 0.001
I_min_ (mm^4^)	0.0043 ± 0.0004	0.0040 ± 0.0005	0.0048 ± 0.0004	0.0040 ± 0.0002

Data are presented as the mean ± standard error

*BMD* bone mineral density, *BV/TV* ratio of bone volume to total volume, *HA* hydroxyapatite, *J* inertia, *I*_*max*_ maximum second moment of inertia, *I*_*min*_ minimum second moment of inertia

**Fig. 3:**
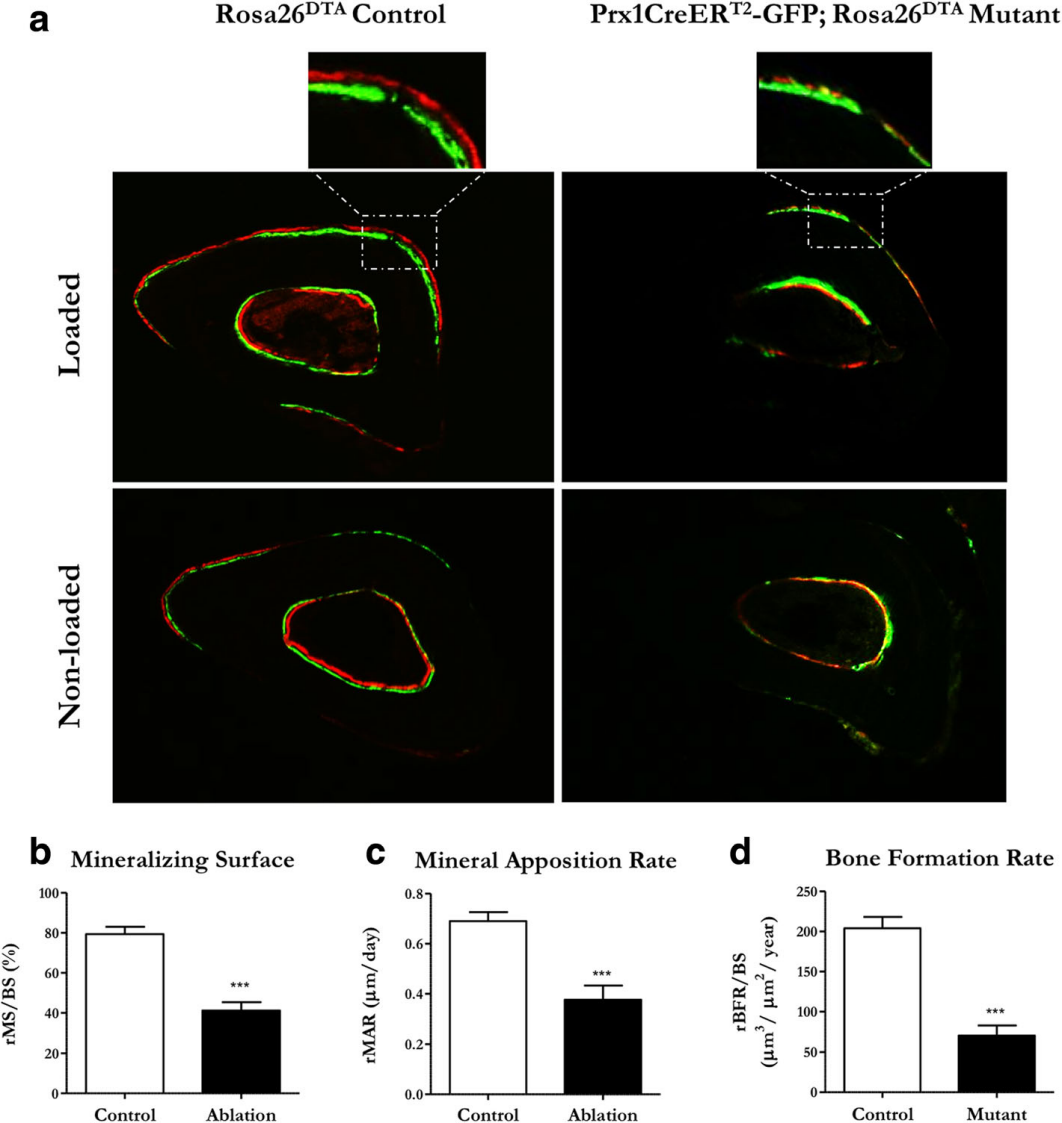
Mineralization and load-induced bone formation are severely attenuated in mice lacking OCPs. Skeletally mature Rosa26^DTA^ control and Prx1CreER-GFP;Rosa26^DTA^ ablation animals injected with tamoxifen were exposed to ulnar loading and the resulting mineralizing surfaces were labeled with calcein (green) and alizarin (red) fluorochrome dyes. Mice lacking periosteal OCPs demonstrated poor mineralization, indicated by a lack of labeling at the periosteal surface in both loaded and nonloaded ulnae (**a**). We performed dynamic histomorphometry and confirmed this visual observation (**b**). Ablated animals also exhibited an inferior mineral apposition rate (**c**), resulting in attenuated bone formation compared with controls (**d**). Loaded ulnae were normalized to nonloaded contralateral limbs. Micrographs were collected at 10X. Data are reported as mean and standard error. *n* = 16 for each group, ***p < 0.0001. rBFR/BS relative bone formation rate/bone surface, rMAR relative mineral apposition rate, rMS/BS relative mineralizing surface/bone surface

We then exposed skeletally mature adult mice to compressive axial ulnar loading and visualized fluorochrome labels approximately 2 weeks following loading to assess mineralization with standard cage activity and in response to load. Control animals demonstrated some mineralization in the nonloaded limb and, as expected, the mineralizing surface was greater in response to load (Fig. 3). We also observed a distinct gap between the alizarin and calcein labels in loaded control animals, indicating newly formed bone. In contrast, ablation animals demonstrated very little mineralizing surface under nonloaded conditions and a weak increase in response to load, suggesting very little bone was formed under static and loaded conditions. We quantified our observations via dynamic histomorphometry and, indeed, mutants lacking OCPs have a smaller mineralizing surface (Fig. 3b) and decreased mineral apposition rate (Fig. 3c). Consequently, mice lacking OCPs have a severely attenuated bone formation rate (Fig. 3d).

We then performed H&E stains to visualize any potential abnormalities in bone tissue in the loaded ulna of animals with and without periosteal OCPs (Additional file 1: Figure S1). Surprisingly, the periosteum was consistently thinner in ablated animals, perhaps due to loss of OCPs in the cambium layer. We initially noticed this trend when we confirmed the ablation model and noted that the periosteum appeared thinner when GFP^+^ cells were absent (Fig. 2b). We speculated that atypical woven bone may have formed in mutants, but histology revealed that both groups generated normal lamellar bone in response to load. Interestingly, we identified periosteal cells differentiating into osteoblasts to lay down new matrix in response to load in control animals, but this behavior was lost entirely in the ablation animals.

### Primary periosteal progenitors have an osteogenic response to mechanical stimulation

**Fig. 4:**
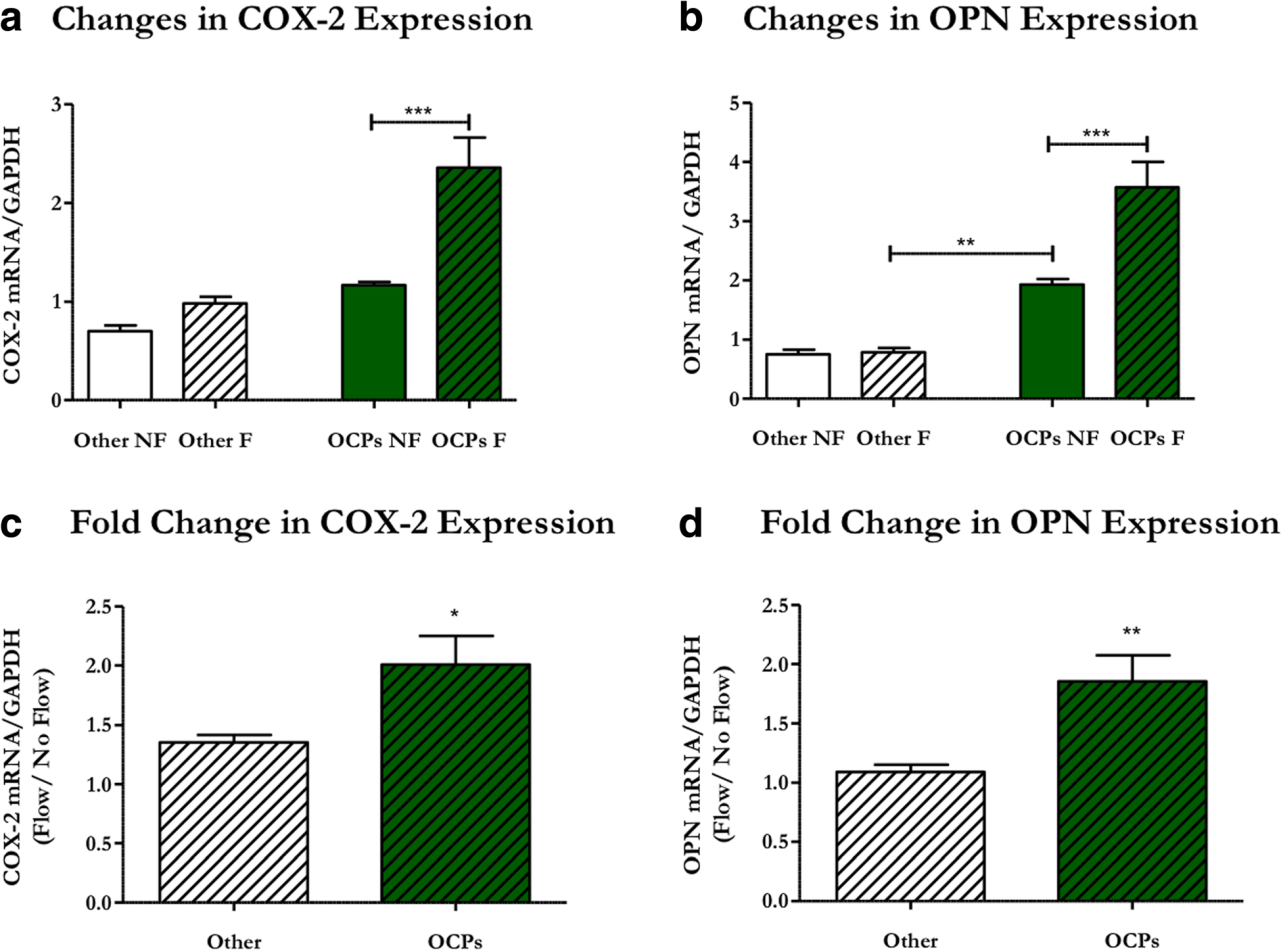
Periosteal OCPs uniquely respond to mechanical stimulation in an osteogenic manner. Primary periosteal cells isolated from 3-week-old Prx1CreER-GFP mice were exposed to 1 h of oscillatory fluid flow (OFF) and changes in mRNA expression were quantified via RT-qPCR. Prx1-expressing OCPs (green) exposed to OFF (line pattern) demonstrated increases in Cyclooxygenase-2 (COX-2) (**a**) and Osteopontin (OPN) expression (**b**) compared with controls, whereas other cells of the periosteum (white) were nonresponsive. OCPs have elevated OPN expression compared with other cells of the periosteum under static conditions (**b**). Additionally, the fold changes in COX-2 (**c**) and OPN (**d**) expression were significantly higher for OCPs compared with other cells of the periosteum. mRNA expression was normalized to GAPDH expression. Data are reported as mean and standard error. Other no-flow *n* = 5, other flow *n* = 7, OCP no-flow and flow *n* = 6, *p < 0.01, ***p < 0.0001

Although periosteal tissue [25] and calvaria periosteal osteoprogenitors [26] respond to physical stimulation, it is unknown whether OCPs in long bone periosteal tissue are mechanoresponsive. We therefore isolated cells from murine tibial periosteum and exposed them to oscillatory fluid flow (OFF) to determine if these cells respond to mechanical stimulation. Furthermore, we separated periosteal Prx1-expressing OCPs from the other cells of the periosteum to evaluate whether this population has a greater osteogenic response to physical stimuli. Indeed, sorted periosteal OCPs exposed to OFF exhibited increased COX-2 mRNA production compared with static controls (Fig. 4a), indicating a cellular reaction to physical stimulation. OCPs also demonstrated a flow-induced increase in OPN, which indicates an osteogenic response to fluid flow (Fig. 4b). The OCP response to flow is unique since other cells of the periosteum demonstrated no change in COX-2 or OPN expression (Fig. 4a, b). Interestingly, Prx1-expressing OCPs had significantly higher levels of OPN mRNA under static conditions than other cells of the periosteum in static (*p* < 0.05) or flow conditions (Fig. 4b). Periosteal OCPs also exhibited significantly greater fold changes in COX-2 and OPN expression compared with other cells of the periosteum (Fig. 4c, d), indicating that OCPs are drastically more responsive to mechanical stimulation. Collectively, these data suggest periosteal OCPs directly sense mechanical loading and respond in a pro-osteogenic manner.

### Primary cilia are necessary for the osteogenic response to physical stimulation

**Fig. 5:**
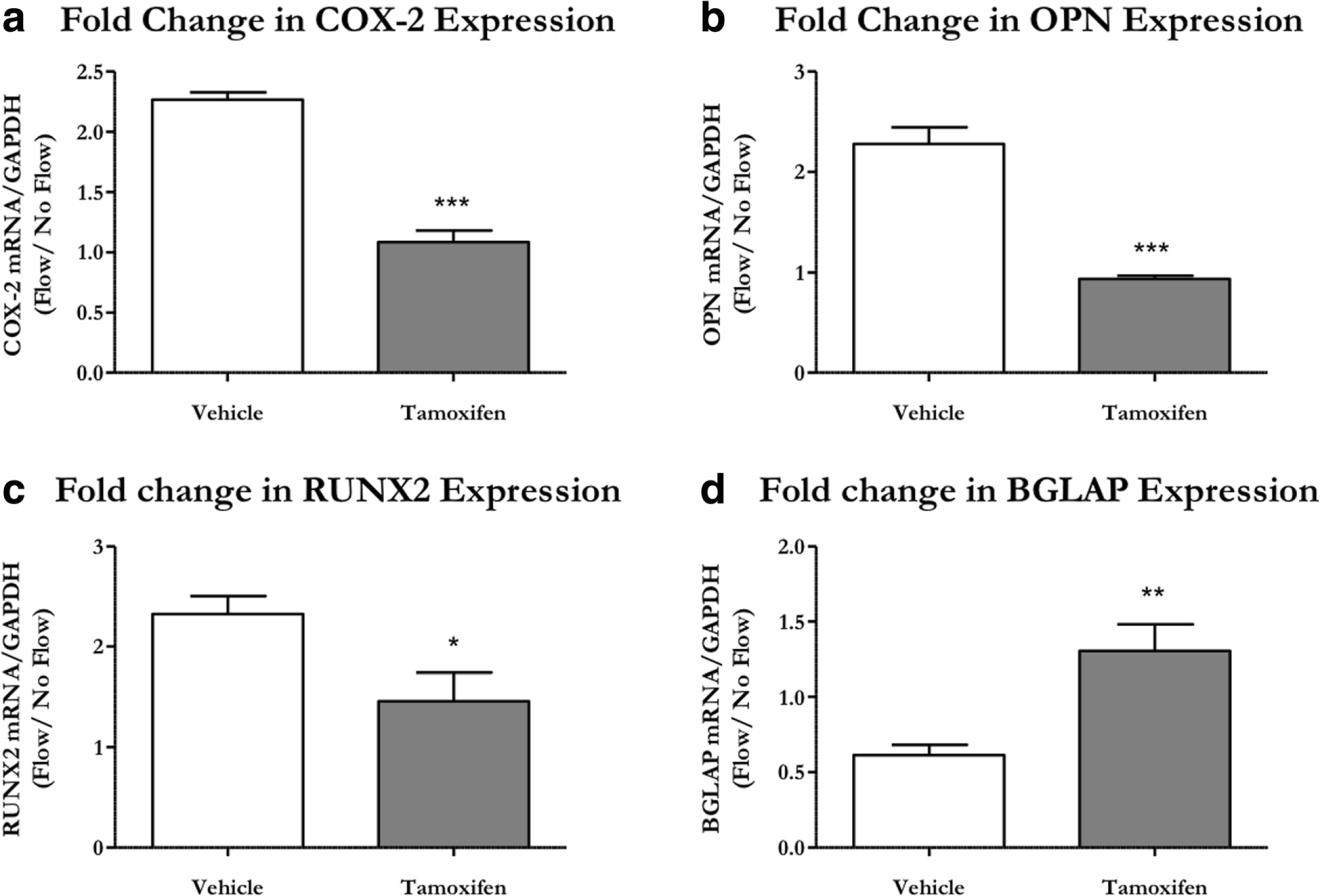
OCP primary cilia are necessary for the flow-induced osteogenic response. Primary periosteal OCPs isolated from 3-week-old Prx1CreER-GFP;Ift88^fl/fl^ mice were treated with the active compound of tamoxifen or vehicle control and exposed to 1 h of oscillatory fluid flow (OFF). Vehicle control OCPs (white) demonstrated an increase in Cyclooxygenase-2 (COX-2) (**a**), Osteopontin (OPN) (**b**), and Runt-related transcription factor 2 (RUNX2) (**c**) expression, but a decrease in Bone gamma-carboxyglutamic acid-containing protein (BGLAP) (**d**) expression. These effects were lost in OCPs treated with tamoxifen (gray) to disrupt ciliogenesis. mRNA expression was normalized to GAPDH expression and OFF samples were normalized to static controls. Data are reported as mean and standard error. Vehicle *n* = 6, tamoxifen *n* = 5, ***p < 0.0001

We then examined whether this osteogenic response to flow was mediated by the primary cilium. Primary periosteal OCPs were isolated from Prx1CreER-GFP;Ift88^fl/fl^ animals and treated with a vehicle control or the active metabolite of tamoxifen to induce a primary cilium knockout. We evaluated mRNA expression levels of COX-2, which indicates a cellular response to physical stimulation, and OPN, RUNX2, and BGLAP, which are markers for osteogenesis. OCPs with intact cilia demonstrated increased COX-2 (*p* < 0.001), OPN (*p* < 0.05), and RUNX2 (*p* < 0.001), but decreased BGLAP (*p* < 0.05) expression in response to flow. These flow-induced changes in mRNA expression were absent in KO cells, indicating an abrogated response to OFF (Fig. 5). This suggests that primary cilia are necessary for periosteal OCPs to 1) sense mechanical stimulation and 2) respond to fluid shear in an osteogenic fashion.

### Periosteal progenitors also respond to paracrine signals from stimulated osteocytes through a cilium-mediated mechanism

**Fig. 6:**
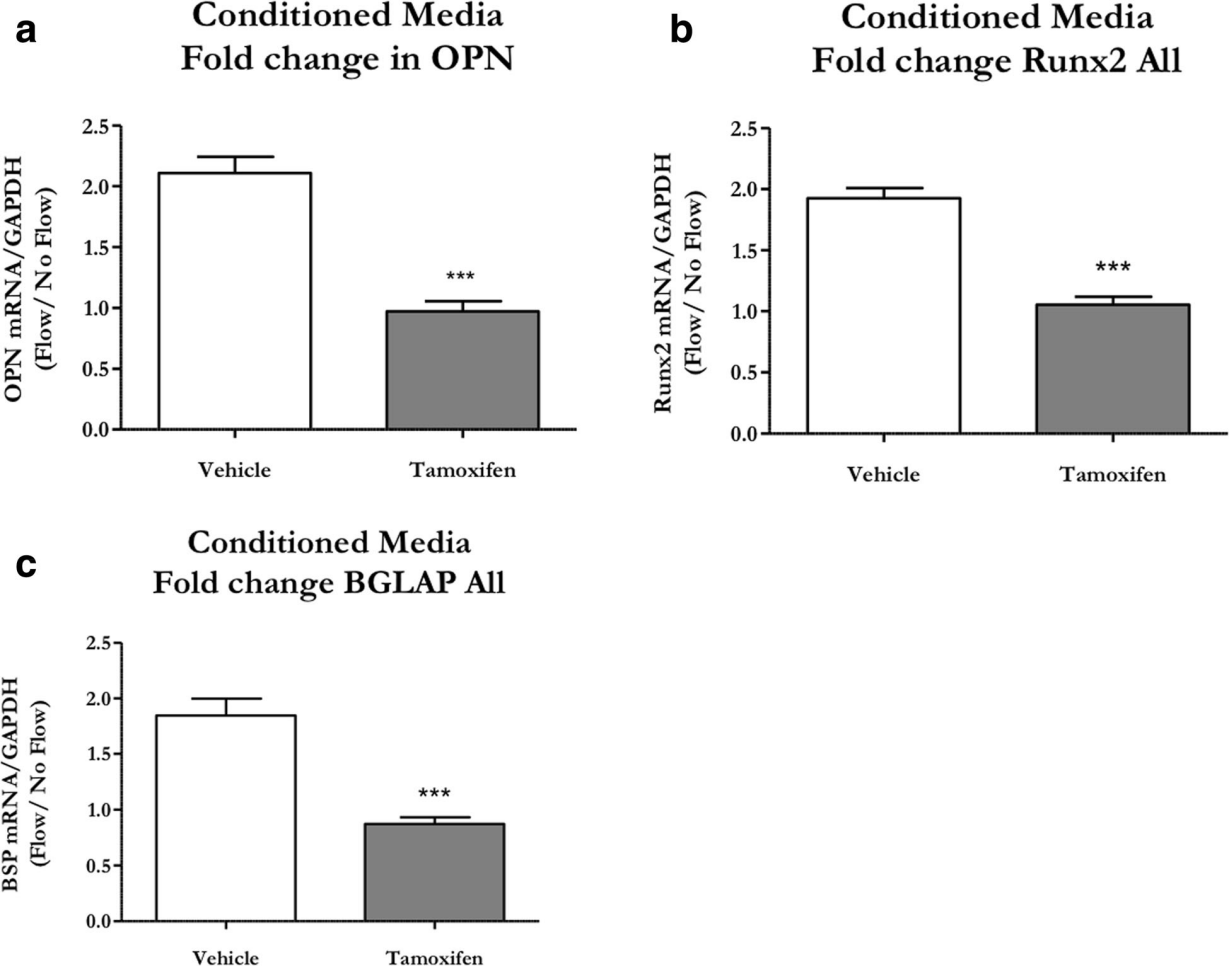
OCPs require primary cilia to respond to paracrine signals from mechanically stimulated osteocytes. Primary periosteal OCPs isolated from 3-week-old Prx1CreER-GFP;Ift88^fl/fl^ mice were treated with the active compound of tamoxifen to disrupt ciliogenesis or vehicle control and then treated with conditioned media from MLO-Y4s exposed to oscillatory fluid flow (OFF) or static controls. Vehicle control OCPs (white) demonstrated a nearly twofold increase in Osteopontin (OPN) (**a**), Runt-related transcription factor 2 (RUNX2) (**b**), and Bone gamma-carboxyglutamic acid-containing protein (BGLAP) expression (**c**). This effect was lost in OCPs treated with tamoxifen (gray). mRNA expression was normalized to GAPDH expression and OFF samples were normalized to static controls. Data are reported as mean and standard error. *n* = 5 for both groups, ***p < 0.0001

Thus far, we have shown that bone-forming osteoblasts arise from periosteal OCPs in response to physical stimulation. Another mechanism by which cells may differentiate into osteoblasts is through paracrine signaling from mechanically stimulated osteocytes [8]. We explored this possibility through a conditioned media study. MLO-Y4 osteocyte-like cells were exposed to OFF and the resulting media was transferred onto periosteal OCPs with and without primary cilia. Indeed, OCPs with intact cilia demonstrated an increase in mRNA expression of the osteogenic markers OPN (*p* < 0.05), RUNX2 (*p* < 0.05), and BGLAP (*p* < 0.01). Again, the fold changes in mRNA expression were lost with cilium disruption (Fig. 6), indicating a loss of paracrine signaling.

## Discussion

Despite its ubiquity in embryonic development, Prx1 expression is restricted to the periosteum in the adult ulna. Prx1 expression is rampant in the mesenchymal limb bud but it has been incompletely characterized after birth [11]. Previous studies report labeled cells in a wide variety of tissues, suggesting the Prx1Cre transgene denotes a multipotent mesenchymal progenitor population [14]. However, these experiments utilize constitutively active models which label cells that have expressed Prx1 at any point in time, including embryonic development. Prx1-expressing cells and their progeny populate a vast array of tissues during development, so it is perhaps not surprising that the authors identified labeled cells throughout the adult skeleton. These constitutive models therefore successfully track Prx1-expressing cells and their progeny, but do not outline specific Prx1 expression patterns in the adult skeleton. Kawanami et al. [11] developed a tamoxifen-inducible model containing Prx1-driven GFP expression and suggested that Prx1 expression is perhaps more confined in the limb after birth. We crossed this inducible model with a red fluorescent reporter and visualized GFP and tdTomato in our Prx1CreER-GFP;Rosa26^tdTomato^ model to more accurately identify cells that express Prx1 in the forelimbs of skeletally mature adults. The vast majority of labeled cells were found in the periosteum, with some present in the perichondrium and shoulder tendon, but were absent from bone marrow, muscle, and bone. The perichondrial expression may be unique to mice since their epiphyseal growth plates do not close [27] like those in other animals. Due to physical distance alone, it is highly unlikely that cells from the perichondrium or shoulder tendon would contribute to bone formation in the midshaft of the diaphysis. Prx1-expressing cells isolated from periosteal tissue demonstrate an osteochondrogenic nature in vitro [11]; therefore, we are confident that our Prx1 model is an excellent tool for evaluating the role of periosteal OCPs in load-induced bone formation. This is in agreement with a recent fracture study concluding that the Prx1 model was appropriate for studying a unique population of self-regenerating periosteal cells in older mice [12].

Prx1-expressing periosteal OCPs are known to dominate fracture repair but our results demonstrate they also contribute to adult bone formation to a great degree. Skeletal trauma elicits a response from many cell types, so it is perhaps not surprising that Prx1-expressing cells participate in fracture repair. Load-induced bone formation is a relatively milder response to a less intense external stimulus and involves differentiation of osteogenic precursors to form new bone via intramembranous ossification. Our dynamic histomorphometry data indicate that load-induced bone formation is almost entirely lost when Prx1-expressing periosteal OCPs are absent. Although this outcome is somewhat expected since cambium layer progenitors are known to participate in intramembranous ossification, these results are important for three reasons. First, our data contribute to mounting evidence that Prx1-expressing cells play a critical role in adult skeletal maintenance. All mesenchymal cells in the limb bud express Prx1 and therefore have been heavily investigated in embryonic development, but Prx1-expressing cells are largely unexplored in adult skeletal phenomena. Our results, combined with studies showing that Prx1-expressing cells populate the fracture callus [11, 12, 28], indicate these OCPs continue to influence skeletal metabolism and morphology following their well-documented initial role in embryonic skeletal development. Second, the extreme impact of ablating Prx1-expressing periosteal OCPs suggests these osteogenic precursors are perhaps the primary source of osteoblasts that form new bone in response to heightened physical loads. This population has also been implicated as the main cell source for the fracture callus [12, 29] and overall appears to be a critical supply of osteogenic precursors. Lastly, mutants still display some mineralization, suggesting that Prx1-expressing cells are not the only source of bone-forming osteoblasts. The mineralization that does occur in mutants is potentially from other osteogenic precursors in the periosteum or bone lining cells, which line the periosteal surface and are a continuous source of osteoblasts in mature bone [30, 31]. Another possibility is that some OCPs remained due to incomplete Cre recombination, but we found that Prx1-expressing cells were totally ablated in vivo in our model (Fig. 2b). Regardless of other cell involvement, periosteal OCPs contribute to adult skeletal maintenance to such a degree that they deserve more consideration for adult bone tissue regeneration strategies.

For the first time, we have shown that periosteal OCPs have an osteogenic response to direct physical stimulation, as well as paracrine signals from mechanically stimulated osteocytes. Furthermore, we identified that the primary cilium is a major factor in periosteal OCPs sensing stimulation and inducing osteogenesis. We previously identified that load-induced bone formation was attenuated in animals containing a Prx1-driven conditional primary cilium knockout (Chen et al., manuscript submitted); however, the Prx1-expressing periosteal OCP primary cilium’s exact role during bone formation remained unclear. One possibility is that periosteal OCPs directly sense mechanical stimulation via their primary cilia and consequently differentiate into bone-forming osteoblasts. Indeed, we found that periosteal OCPs have an immediate osteogenic response to fluid shear that is nearly abrogated when cilia are absent. An earlier study utilized Northern blotting to determine that OPN and BGLAP mRNA expression in rat tibia periosteum is decreased 2 h after exposure to four-point bending [32]. The authors also found increased expression of proliferation markers and, combined with the decrease in mRNAs encoding bone matrix proteins, concluded that the initial periosteal response to load is rapid proliferation. We also observe a decrease in BGLAP, which suggests proliferation, but a contradictory increase in OPN, which favors osteogenic differentiation. However, we examined a specific OCP cell population whereas the aforementioned authors quantified mRNA expression in whole periosteal tissue. We speculate that the Prx1-expressing population possesses unique osteogenic characteristics and may simultaneously proliferate and differentiate. A second theory is that osteocytes, which are known to detect mechanical loading and activate osteogenic precursors [8], signal to periosteal OCPs through a primary cilium-mediated mechanism to encourage osteoblastic differentiation and subsequent bone formation. Interestingly, periosteal OCPs treated with conditioned media from mechanically stimulated osteocytes demonstrate increased mRNA expression of osteogenic markers 48 h later. We suspect the increase in BGLAP observed here (Fig. 6c), which is contradictory to our OFF results (Fig. 5d), is due to a discrepancy in the evaluated time points. For example, osteoblasts exposed to fluid shear in vitro do not demonstrate an increase in BGLAP expression until 2 h after exposure [33]. Similarly, we did not find changes in COX-2 expression after 48 h since this is an immediate, short-term response. We therefore propose that, during load-induced bone formation, periosteal OCP primary cilia directly sense loading and receive signals from mechanically stimulated osteocytes. These stimuli then induce mechanosensitive OCP differentiation into active osteoblasts, as well as rapid proliferation of OCPs, which later differentiate to form bone.

Although osteogenesis is severely attenuated in its absence, the primary cilium is not the only mechanism by which OCPs sense and respond to stimulation. The cell membrane alone is capable of transducing physical stimuli via mechanosensitive ion channels, cadherins, integrins/ focal adhesions, purinergic receptors, and connexins [34]. For example, TRPV4 is a calcium ion channel found in the primary cilium and cell membrane and is known to be important for osteocyte mechanotransduction. We previously identified that disrupting TRPV4 attenuates flow-induced osteogenesis when the cilium is intact [35], and so it is possible that TRPV4 in the cell membrane responds to physical stimulation independent of the primary cilium. The primary cilium is also not uniquely involved in paracrine signaling since a variety of receptor/ligand interactions exist independently of the cilium. Paracrine signals from osteocytes are known to influence osteogenic differentiation [36], but specific pathways have yet to be characterized. One possibility is that IGF, which is secreted by osteocytes, encourages OCP differentiation [37, 38]. Additionally, FGF, Wnt, Hedgehog, and TGFβ/BMP signaling pathways involve receptor/ligand interactions and are strong candidates for OCP differentiation. Interestingly, the primary cilium is associated with these pathways [39], and so we speculate that the cilium mediates receptor/ligand interactions to some degree. More work is required to fully understand osteocyte-OCP signaling, but we hypothesize that the primary cilium works in concert with other receptor/ligand interactions at the cell surface to generate a complete response. For these reasons, osteogenesis is not completely lost with primary cilium deletion in our fluid shear and conditioned media in-vitro studies. However, the drastic attenuation we observe does suggest the primary cilium plays a predominant role.

Load-induced bone formation decreases as mice age, despite evidence suggesting that periosteal OCPs maintain function independently of age. In young adult mice, periosteal progenitors are the main contributor to fracture callus formation [28] and our data suggest they also play a significant role in adult bone formation. Although 16-week-old mice contain a wealth of periosteal progenitors, it is believed that older mice possess markedly fewer periosteal cells and, consequently, have attenuated load-induced bone formation. One study found that 19-month-old mice had significantly fewer periosteal cells in response to loading compared with younger 16-week-old mice, resulting in severely limited bone formation [40]. Interestingly, the initial number of periosteal cells prior to loading was similar between the groups, suggesting a lack of proliferation and differentiation in older mice. In another study, 54-week-old mice displayed inferior fracture repair compared with 8-week-old mice, but repair was enhanced in both groups when PTH 1–34 was intermittently administered [41]. The authors therefore concluded that younger periosteal cells are able to produce more matrix and differentiate more rapidly. However, other studies indicate that periosteal progenitors consistently proliferate and function independent of age [42–44]. In fact, a recent study demonstrated that Prx1-expressing periosteal cells repopulate the periosteum after fracture and continue to be the dominant cell type in the fracture callus in adult mice, even after multiple fractures are initiated [12]. Since the periosteum contains a variety of cells, one possibility is that the number of OCPs decreases with age, while the total number of periosteal cells remains comparable. Bone marrow stromal cells from young and old human donors form similar amounts of mineralized matrix in vitro and bone in vivo, indicating that cell function remains intact with age [45]. This further supports the idea that decreased bone formation in older age is a consequence of decreased proliferation and fewer active osteoblasts. More importantly, it suggests that progenitors, regardless of age, may be expanded in vitro to provide a sufficient number of cells for tissue regeneration. Another explanation for decreased bone formation with age is a reduction in sensitivity to mechanical stimulation [46, 47]. Studies indicate older mice and rats are unresponsive to low strains that successfully trigger apposition in young mice [46, 48, 49], and one group suggests bone formation and resorption, as dictated by strain, becomes dysregulated [49]. However, it is unclear whether the decreased sensitivity results from changes at the tissue, cellular, and/or molecular levels. Overall, the mounting evidence indicates that strategies to produce bone at any age will need to generate a sufficient number of OCPs and/or enhance OCP function.

Primary OCPs were easily extracted from periosteal tissue and expanded in culture for our in-vitro experiments, suggesting they are an attractive source for tissue regeneration strategies. A typical isolation was performed with 6–8 juvenile mice and periosteal tissue was extracted from only the ulna and tibia. Cells adhered to the dish surface within 3–5 days and continued to migrate from tissue for as long as 2 weeks after the dissection. Within 3 weeks of the initial dissection, approximately 8 million cells were available for sorting. Interestingly, only 3.3 ± 0.8% (*n* = 10 sorts) of the 8 million cells were GFP^+^ Prx1-expressing OCPs. This finding is perhaps surprising considering that Prx1-expressing cells make up such a small percentage of the total cells in the periosteum, yet have such a drastic impact on adult bone formation and fracture repair. Similarly, in the aforementioned repeated fracture study, the authors identified very few self-renewing Prx1-expressing cells in new periosteum following fracture, but these rare cells still made up the vast majority of callus cells in subsequent fractures [12]. Despite the low percentage and some cell death due to sorting, these cells rapidly proliferate within 24 h and we were able to generate enough cells for a typical fluid flow study (about 500 k) within a week. These cells continued to proliferate at the same rate and were passaged up to 10 times spanning 2 months after sorting. The percentage of Prx1-expressing cells increased when given more time in culture before sorting, suggesting these OCPs proliferate at a higher rate than other cells of the periosteum. This extraction is faster or similar to the amount of time it takes to isolate primary MSCs [50]. Moreover, MSCs require further guidance in vitro to differentiate into osteoblasts, whereas periosteal OCPs are preprogrammed towards an osteoblastic or chondrogenic lineage and therefore require less culture time [11, 51]. Further work must be done to compare human periosteal progenitors and MSCs to obtain a definitive answer but, minimally, our preliminary observations suggest that the periosteum is a promising source for osteogenic precursors that can be expanded for bone regeneration.

Manipulating the primary cilium is a potential mechanism to enhance periosteal OCP function. In our previous study, mice containing a Prx1-driven cilium knockout formed less bone in response to ulnar loading (Chen et al., manuscript submitted). Specifically, mutants had attenuated mineralization rates, indicating that the primary cilium is important for matrix deposition by OCP-derived osteoblasts. Thus, restoring or enhancing periosteal primary cilium function is critical for periosteal progenitors to proliferate and differentiate during bone formation. The primary cilium is thought to serve as a signaling nexus for the cell and is known to mediate several pathways critical to cell function. For example, the cilium was recently found to regulate TGF-β signaling [52, 53], which is important for periosteal progenitor proliferation and differentiation [54], bone matrix protein synthesis [55], and load-induced bone formation [32]. The primary cilium also mediates Wnt signaling, a pathway critical to osteoblast lineage commitment and skeletal homeostasis [56]. In the aforementioned study, Yukata et al. determined that intermittent PTH 1–34 treatment stimulated Wnt signaling, increasing the number of participating periosteal progenitor cells in the fracture callus [41]. We speculate that if the primary cilium is potentiated, its associated signaling pathways will be enriched. Indeed, we recently demonstrated that pharmacologically lengthening osteocyte primary cilia in vitro enhances their mechanosensitivity and, consequently, their osteogenic response to mechanical stimulation [57]. This mechanism has been confirmed in other cell types [58], suggesting that potentiating cilia generally enhances the function of cells, including periosteal progenitors. More importantly, this pharmacological treatment was able to recover cilium structure and sensitivity in cells with impaired cilia, making it a viable option for stimulating older progenitor cells.

## Conclusion

Collectively, our results encourage the use of periosteal Prx1-expressing OCPs and manipulation of their primary cilia for tissue engineering and regenerative medicine (TERM) applications. The mechanisms for adult bone formation are highly similar between mice and humans and so our work provides important insights for potential therapeutics. The current preferred method for bone TERM in patients is to implant a scaffold seeded with MSCs that have been extracted and guided to an osteoblastic lineage in vitro [6]. Periosteal progenitors are potentially better suited for bone tissue regeneration because they preferentially provide both osteoblasts and chondrocytes [28]. Our studies demonstrate that periosteal OCPs are prevalent and active in skeletally mature adult mice. More importantly, we identified that periosteal OCPs are not only inherently involved in, but are perhaps the dominant contributor to, load-induced adult bone formation. This importantly builds upon recent work demonstrating that periosteum-derived progenitors are the most prevalent cell source in the fracture callus and uniquely dominate repair after multiple fractures [28]. This suggests periosteal OCPs have a surprising ability to proliferate and generate new bone in vivo, even though they represent a mere 3% of cells found in the periosteum. Additionally, OCPs are easier to extract from the subcutaneous periosteum in several locations compared with MSCs, and can be expanded in culture in vitro. We also found that primary cilia are an important contributor to the osteogenic nature of OCPs. OCP primary cilia are therefore attractive therapeutic targets since sensory organelles can be manipulated in vitro to enhance osteogenesis [57, 58]. Overall, this Prx1-expressing periosteal OCP population satisfies the criteria for a much-desired progenitor source and should be considered a strong contender for future research and clinical applications in skeletal tissue regeneration.

## Additional file

Additional file 1: **Figure S1.** Mice lacking OCPs lack load-induced osteoblast differentiation at the periosteal surface. H&E stains of tissue sections from control (left) and experimental animals (right). Control animals exhibited differentiating osteoblasts at the periosteal surface (left, black box). These differentiating osteoblasts were not observed in animals with ablated OCPs (right) or the nonloaded contralateral limbs of both groups (data not shown). Micrographs were collected at 20X magnification. (TIF 7918 kb)

## Abbreviations

BGLAP: Bone gamma-carboxyglutamic acid-containing protein
BMD: Bone mineral density
BV/TV: Ratio of bone volume to total volume
COX-2: Cyclooxygenase-2
CS: Calf serum
CT: Computed tomography
DTA: Diphtheria toxin A
FBS: Fetal bovine serum
GAPDH: Glyceraldehyde 3-phosphate dehydrogenase
GFP: Green fluorescent protein
H&E: Hematoxylin and eosin
KO: Knockout
MEM: Minimum essential medium
MSC: Mesenchymal stem cell
OCP: Osteochondroprogenitor
OFF: Oscillatory fluid flow
OPN: Osteopontin
P/S: Penicillin/streptomycin
PBS: Phosphate-buffered saline
PCR: Polymerase chain reaction
Prx1: Paired related homeobox 1
rBFR/BS: Relative bone formation rate/bone surface
rMAR: Relative mineral apposition rate
rMS/BS: Relative mineralizing surface/bone surface
RUNX2: Runt-related transcription factor 2
TERM: Tissue engineering and regenerative medicine
