# Correction to: Periosteal progenitors contribute to load-induced bone formation in adult mice and require primary cilia to sense mechanical stimulation

**DOI:** 10.1186/s13287-018-0975-1

**Published:** 2018-08-28

**Authors:** Emily R. Moore, Ya Xing Zhu, Han Seul Ryu, Christopher R. Jacobs

**Affiliations:** Columbia University Department of Biomedical Engineering, 500 W 120th St, New York, NY 10027 USA

## Correction

The original article [1] contained two minor errors affecting the labelling of Fig. 3d and Figs. 6b & 6c; these errors have now been corrected in the respective figures in the original article.
