# Supplementary figures and images for: Periosteal progenitors contribute to load-induced bone formation in adult mice and require primary cilia to sense mechanical stimulation
Source: Stem Cell Res Ther. 2018 Jul 11;9:190. doi: 10.1186/s13287-018-0930-1 (PMC6042447; doi:10.1186/s13287-018-0930-1)

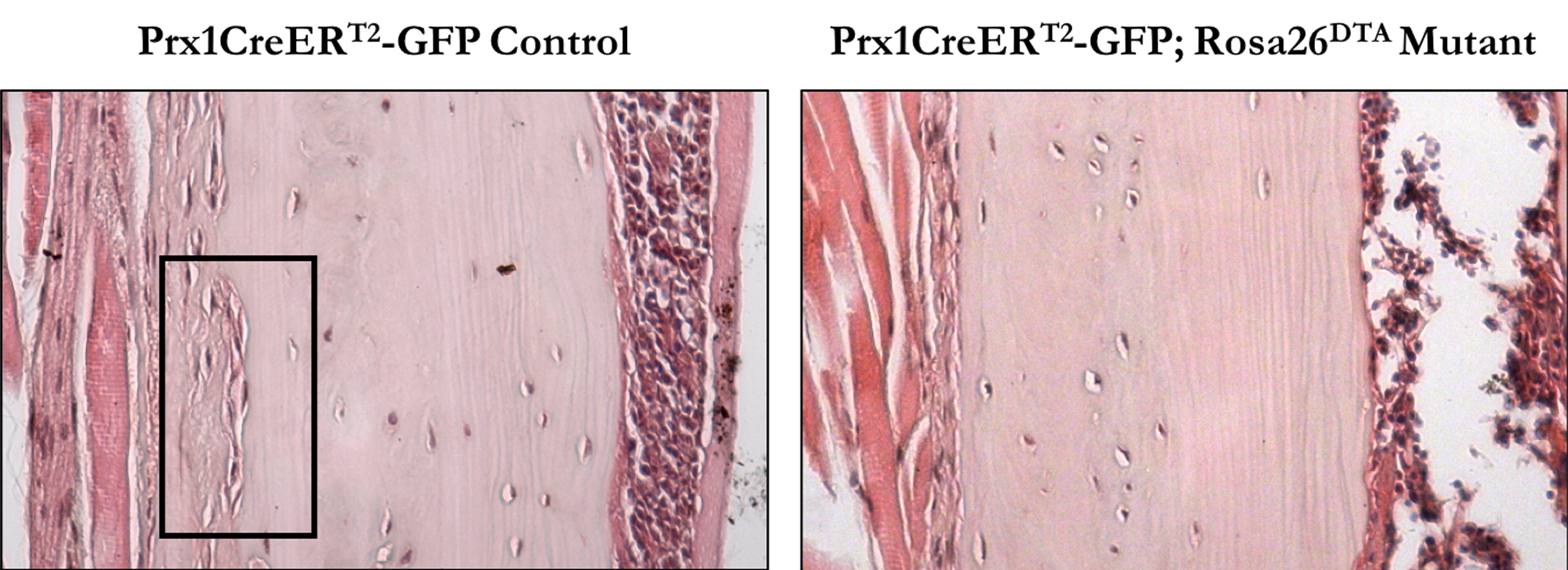

Supplement: Supplementary file 1 — Figure S1. Mice lacking OCPs lack load-induced osteoblast differentiation at the periosteal surface. H&E stains of tissue sections from control (left) and experimental animals (right). Control animals exhibited differentiating osteoblasts at the periosteal surface (left, black box). These differentiating osteoblasts were not observed in animals with ablated OCPs (right) or the nonloaded contralateral limbs of both groups (data not shown). Micrographs were collected at 20X magnification. (TIF 7918 kb) [file 13287_2018_930_MOESM1_ESM.tif]
